# The association between day-to-day stress experiences and work–life interference among office workers in academia: an ecological momentary assessment study

**DOI:** 10.1007/s00420-022-01915-y

**Published:** 2022-09-15

**Authors:** Larissa Bolliger, Gillian Debra, Junoš Lukan, Rani Peeters, Elena Colman, Ellen Baele, Mitja Luštrek, Dirk De Bacquer, Els Clays

**Affiliations:** 1 Department of Public Health and Primary Care, Ghent University, 9000 Ghent, Belgium; 2 Department of Developmental, Personality, and Social Psychology, Ghent University, 9000 Ghent, Belgium; 3 Department of Intelligent Systems, Jožef Stefan Institute, Jožef Stefan International Postgraduate School, 1000 Ljubljana, Slovenia

**Keywords:** Academia, Day-to-day work experiences, Ecological momentary assessment (EMA), Job strain, Office workers, Work-life interference

## Abstract

**Purpose:**

We investigated relations between day-to-day job demands, job control, job strain, social support at work, and day-to-day work–life interference among office workers in academia.

**Methods:**

This study is based on a 15-working day data collection period using an Ecological Momentary Assessment (EMA) implemented in our self-developed STRAW smartphone application. We recruited office workers from two academic settings in Belgium and Slovenia. Participants were repeatedly asked to complete EMAs including work stressors and work interfering with personal life (WIPL) as well as personal life interfering with work (PLIW). We applied fixed-effect model testing with random intercepts to investigate within- and between-participant levels.

**Results:**

We included 55 participants with 2261 analyzed observations in this study. Our data showed that researchers with a PhD reported higher WIPL compared to administrative and technical staff (β = 0.37, *p* < 0.05). We found significant positive associations between job demands (β = 0.53, *p* < 0.001), job control (β = 0.19, *p* < 0.01), and job strain (β = 0.61, *p* < 0.001) and WIPL. Furthermore, there was a significant interaction effect between job control and social support at work on WIPL (β = − 0.24, *p* < 0.05). Additionally, a significant negative association was found between job control and PLIW (β = − 0.20, *p* < 0.05).

**Conclusion:**

Based on our EMA study, higher job demands and job strain were correlated with higher WIPL. Furthermore, we found associations going in opposite directions; higher job control was correlated with higher WIPL and lower PLIW. Higher job control leading to higher imbalance stands out as a novel result.

## Introduction

Job demands and performance pressure are globally increasing in academic work environments. This change is caused by a growth in student numbers, increased focus on high-quality research, international competition, and reductions in government funding for public universities. Furthermore, pressure to publish results, being limited by temporary work contracts, and combining several roles like teaching and research, contribute to work-related stress in academia (Bell et al. 2012).

Exposure to chronic occupational stress has been associated with a multitude of health-related issues, such as mental health problems, cardiovascular diseases, and musculoskeletal discomfort (Brotman et al. 2007; Salvagioni et al. 2017). Furthermore, existing research has shown that chronic work-related stress can increase the risk of work–family conflicts (Bell et al. 2012; Zaheer et al. 2016). A particular risk factor for work–family conflicts is an imbalance between job demands and job control (Brough et al. 2014; Yusuf 2018). Based on a study among US academics, working overtime is a key factor increasing the risk of work–life conflicts (O’Laughlin and Bischoff 2005). UK academics reporting work–family conflicts tended to be less satisfied with their work, less healthy, and more likely to have seriously considered leaving academia (Kinman 2008). Such an imbalance of work and personal life can affect physical and mental well-being and even lead to depression or burnout (Kornitzer et al. 2006; Brotman et al. 2007). A constructive work–life balance on the other hand fosters job performance, job satisfaction, organizational commitment, and private life satisfaction (Sirgy and Lee 2018). While work–family conflict remains a commonly used term in previously published literature, work–life imbalance or interference are becoming more applicable concepts, due to broader coverage of work and private life domains—beyond traditional family constructs (Brough et al. 2014; Yusuf 2018).

Recent research has adopted a more novel perspective on work stress exposures and outcomes, i.e., day-to-day experiences. Such day-to-day work stress—as measured by an Ecological Momentary Assessment (EMA) or similar repeated measures—can lead to decreased overall health and an increase in physical health complaints, such as back pain or fatigue (Piazza et al. 2013). The approach of this paper generated daily information on work-related stress and work–life interference over a prolonged period, providing new and more fine-grained insights into dynamic changes in workers’ stress experiences and outcomes.

Based on our systematic literature review focusing on day-to-day sources and outcomes of work stress, using a repeated measurement design, only two relevant studies assessing work–life interference were identified (Lukan et al. 2022). While these studies looked into work-related stress and work–life interference, they conceptually treated work–life interference merely as an intermediate step in their analysis (Wood et al. 2013; Zhou et al. 2017). In comparison to this previous research, we focused on the association between day-to-day stressors and day-to-day work–life interference as an outcome of such work stress experiences.

Our research aim was to assess whether day-to-day stress experiences impact work–life interference, by investigating the relations between job demands, job control, and job strain and work–life interference among office workers in academia. Additionally, we aimed to assess the role of social support at work in these relations, by investigating whether day-to-day social support levels relate to work–life interference or moderate the association between stress experiences and work–life interference.

## Methods

The reporting of this observational study is based on the STROBE Statement (von Elm et al. 2007). While our STRAW-project included three different data collection methods, this paper is focusing on work experiences collected via EMAs. More detailed information about the STRAW-project can be found in our study protocol paper (Bolliger et al. 2020).

### Study setting, population, and recruitment

Our target population was healthy office workers employed in academic settings. A convenience sample was recruited from two academic organizations in Belgium and Slovenia, using the personal and professional networks of the researchers, internal communication platforms, and printed flyers. In total, 55 office workers were included of which 29 participated in Belgium and 26 participated in Slovenia. Our eligibility criteria were: (1) working at least 80% of a full-time work contract to be sufficiently exposed to work stress, (2) agreeing to install our STRAW application on their personal Android smartphone, (3) agreeing to wear an Empatica wristband continuously from the morning when getting up until the evening when going to bed on working days, and (4) having oral permission from their supervisor to participate in data collection during working hours.

### Study design and procedure

**Fig. 1:**
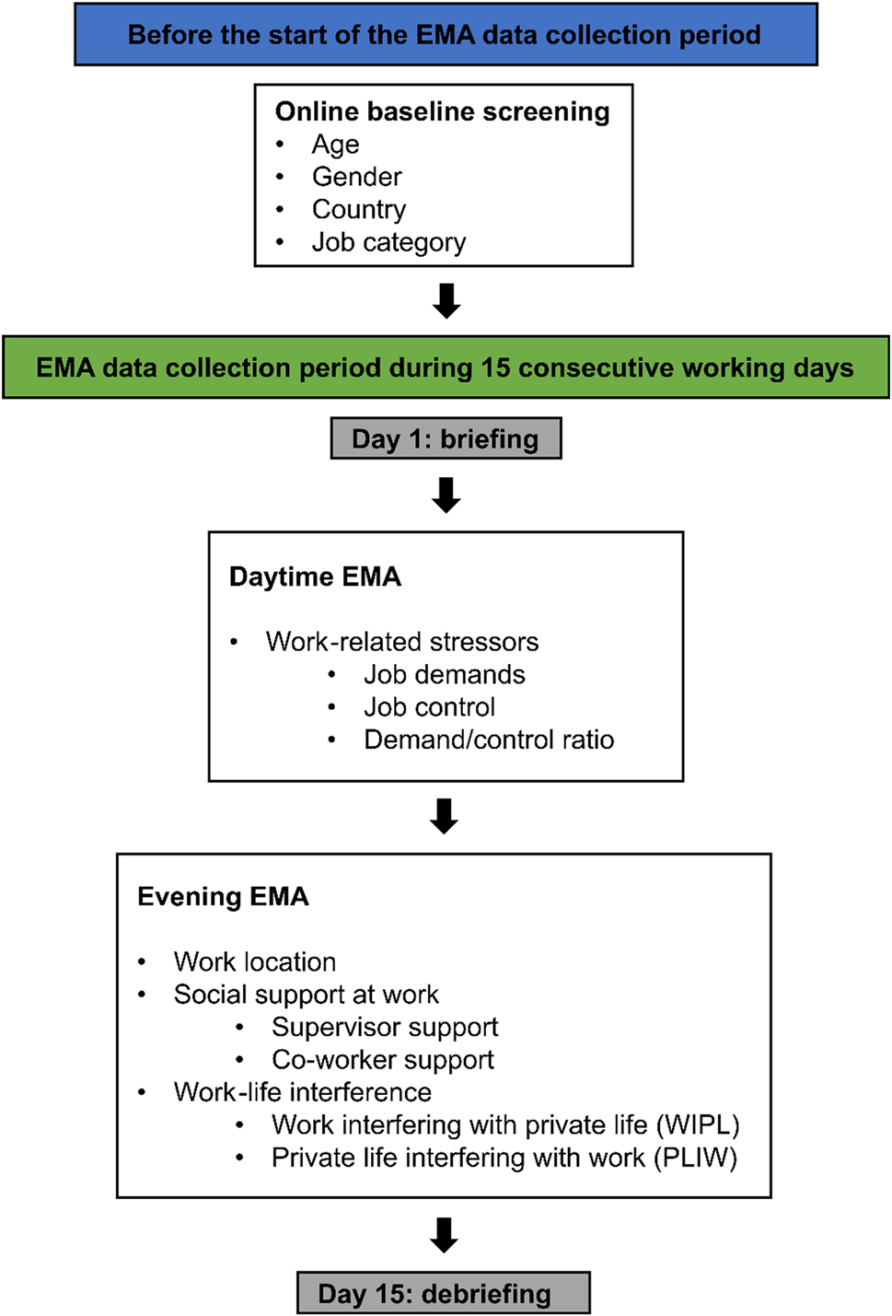
Data collection procedure

The STRAW-project is based on an intensive longitudinal study design using an EMA, implemented in our self-developed STRAW smartphone application (Lukan et al. 2020). This EMA research method enables participants to respond to questionnaires in real time and in their real-world work environments (Bolliger et al. 2020). Our data collection procedure consisted of three phases: (1) online baseline screening and briefing (first day of data collection), (2) EMA data collection period during 15 consecutive working days (excluding weekends), and (3) debriefing (last day of data collection). The data collection procedure is graphically displayed in Fig. 1. Data collection took place from October 2020 until June 2021.

### EMA protocol

In total, ten questionnaires were included in our EMA protocol, focusing on a variety of work-related stressors and stress outcomes. These questionnaires were highly condensed into EMAs of approximately 20 items during the day and 40 items in the evening (Lukan et al. 2021). The order of the questionnaires within the EMAs remained the same throughout the data collection period. Each daytime EMA started with an assessment of the current emotional state (e.g., feeling nervous), followed by questions about the period since the participants started working on that day/since the last questionnaire (e.g., work-related stressors). Each evening EMA started with the question about the work location, followed by a reflection on the whole working day (e.g., social support at work) and questions about the period, since the participants stopped working on that day (e.g., work–life interference). We slightly adapted the wording of the included items to make them suitable to be asked repeatedly.

The EMA protocol was tested during a pilot study with five colleagues in Belgium for 3 consecutive working weeks from February until March 2020. The EMA protocol was originally developed in English and then made available to our participants in Dutch and Slovenian.

Based on our triggering protocol, we implemented a semi-random sampling scheme (Kirtley et al. 2021), meaning that the participants received a smartphone notification prompting an EMA approximately every 90 min during their working day, starting 30 min into their working day at the earliest, until they reported to be done with work for the day. If participants either did not respond to the EMA or swiped it away, they would receive a reminder after 15 min. Participants had the chance to answer the EMA for up to 90 min after the initial triggering before a new EMA would be prompted. Additionally, they received another EMA in the evening at a time of their choosing, when they were typically done with work for the day.

### Measures

#### Work-related stressors

Job demands, job control, and job strain (i.e., demand/control ratio) were included in the daytime EMAs during participants’ working time and were asked repeatedly during the day. We included items of the Job Content questionnaire (Karasek et al. 1998), based on the Job Demand-Control-Support model (Karasek et al. 1998), of which five items focus on job demands and nine items focus on job control. Per EMA, two items out of the five of job demands and two items out of the nine of job control were randomly selected. Exemplary items are: *“My job required working fast”* (job demands) and *“I had a lot of say about what happened on my job”* (job control). Items were answered on a 4-point Likert scale ranging from: *“I strongly disagree (1)”* to *“I strongly agree (4)”*, paired with the introduction: *“Since you started working today/since the last questionnaire”*.

#### Work–life interference

Work interfering with personal life (WIPL) and personal life interfering with work (PLIW) were included in the evening EMAs during participants’ leisure time and were asked once per day. We included seven items focusing on WIPL and four items focusing on PLIW of the Work–Life Balance inventory (Yusuf 2018). Per EMA, two items out of the seven of WIPL and two items out of the four of PLIW were randomly selected. Exemplary items are: *“I miss personal activities because of work”* (WIPL) and *“My work suffers because of my personal life”* (PLIW). Items were answered on a 5-point Likert scale ranging from: *“I strongly disagree (1)”* to *“I strongly agree (5)”*, paired with the introduction: *“Since you stopped working today”*.

#### Social support at work

This variable was included in the evening EMAs during participants’ leisure time and was asked once per day. Social support was measured with four items measuring support from supervisors and four further items measuring support from co-workers from the Job Content questionnaire (Karasek et al. 1998). Per EMA, two items out of the four of supervisor support and two items out of the four of co-worker support were randomly selected. Exemplary items are: *“My supervisor was helpful in getting the job done”* (supervisor support) and *“People I work with were competent in doing their jobs”* (co-worker support). Items were answered on a 4-point Likert scale ranging from: *“I strongly disagree (1)”* to *“I strongly agree (4)”*. We additionally included the option: *“I did not have any contact with my supervisor/co-worker (5)”*. These items were paired with the introduction: *“Referring to your whole working day”*.

#### Additional variables

Work location was included in the evening EMAs during participants’ leisure time and was asked once per day. It was assessed with the following question: *“Where did you do your work?”*, with answer options: *“At the office”*, *“At home”*, *“I moved from between the office and home”*, and *“Other”*. Age, gender, country, and job category were assessed as part of the online baseline screening. The job category was assessed with an open-answer approach.

### Analysis

#### Variables

All scales were averaged over two items per EMA. Since job demands and job control were measured several times during the working day, we calculated the daily means of both. The demand/control ratio was calculated by dividing the daily means of demands by the daily means of control. Social support was included in the analysis first for main effect testing and then as an interaction term, in line with the theory of the Job Demand-Control-Support model (Karasek et al. 1998). We treated supervisor and co-worker support as (general) social support, meaning that we did not differentiate between these two concepts during the analysis. The additional option of *“I did not have any contact with my supervisor/co-worker (5)”* was excluded from the analysis. Work location was included in the analysis as a time-varying covariate. We categorized the results as either *“At home”* or *“Non-home”*. Age (in years), gender, country (Belgium or Slovenia), and job category were included in the analysis as person-level covariates. Job categories were handled as three groups: (1) administrative and technical staff, (2) researchers without a PhD, and (3) researchers with a PhD.

#### Statistical analysis

The initial dataset included 57 participants, 30 in Belgium and 27 in Slovenia. However, one participant withdrew participation after completing the online baseline screening due to a lack of time for further participation. Another person participated throughout the main data collection period, but did not complete the online baseline screening. Therefore, these two participants were excluded from the final dataset, and we analyzed the data of 55 participants. All 55 participants completed the online baseline screening and at least 15 working days of EMA data collection. No participant dropped out between briefing and debriefing. Participant adherence was high with a total of 6639 initiated EMAs, of which 81.0% were completed EMAs. The remaining 14.8% were short indicators such as *“Finished the working day”* and 4.2% were actual incomplete EMAs (Lukan et al. 2021). For this paper, we had 2261 observations that could be included for analysis and 5.2% missing data points (58 observations in WIPL and PLIW and 59 observations in social support).

We included two levels of nested data: repeated assessments per day (level 1) nested within participants (level 2). We tested linear associations between job demands, job control, and job strain (independent variables) and WIPL and PLIW (dependent variables). We decided to handle the subscales of work–life interference as two detached concepts and built up the models separately, one for WIPL and one for PLIW. We focused on fixed-effect model testing, which uses repeated measures within each participant as his or her control. We chose random-intercept modeling over random-slope modeling. First, since we did not aim to model changes over time. Second, we did not assume that the relations between work stress exposures and outcomes would be different between participants. Third, a random-intercept model is more robust for a sample size of 55 participants. To choose our modeling approach, we first created QQ plots and histograms (depicting the distribution of residual terms) to visually inspect our variables and to check the assumptions of normality and homoscedasticity, in which residual terms were plotted against model-predicted values. Second, we used Akaike’s Information Criterion (AIC) for intercept-only models for model comparison, in which the lowest value suggests the best-fitted model. Based on the visual inspection and the model comparison, we opted for the generalized linear mixed models (including random intercepts per participant) with gamma distribution and identity link function (AIC = 5895) over generalized linear mixed models with log-link function (AIC = 5899) and general linear mixed models (AIC = 6317).

In model I, we focused on confounder effect testing, while the covariates were chosen based on comparative literature. For model II, we included our independent variables while dividing it into two sub-models—IIa for job demands and job control and llb for job strain—to avoid multicollinearity. We continued with these sub-models for the rest of the analysis process. For model III, we focused on social support to test as a main effect on work–life interference. In model IV, we included social support as an interaction term, as suggested by previous studies showing an interaction between work-related stressors and social support on stress outcomes.

Analyses were performed using R (version 4.1.0) and RStudio (version 1.4.1717) with statistical significance determined at p < 0.05 (Kuznetsova et al. 2017).

#### Sensitivity analysis

We tested the sensitivity of our results by testing a time effect on day level to see if an increasing or decreasing trend in our dependent variables over 15 days of data collection could be observed. We checked with this analysis for some sort of learning effect over time since the participants started to get used to the EMA content throughout their data collection.

We further tested a weekend effect on our dependent variables to check if a difference between the beginning of the working week (Monday and Tuesday) and the second half of the working week (Wednesday to Friday) could be found. We applied this analysis to investigate if the weekend—characterized by less time spent on work tasks compared to weekdays—had a prolonged effect on our participants’ perception of work–life interference.

## Results

### Descriptive results

The descriptive statistics of our study population are presented in Table 1. The mean age was 34.2 years (SD = 9.7 years) with a range of 24–62 years. As initially planned, we managed to include an approximate balance of men and women (47%) and participants in Belgium and Slovenia (47%). About half of our study population consisted of researchers without a PhD (47%), while the rest consisted of an approximate balance of administrative and technical staff and researchers with a PhD. Table 1 shows the results of the time-varying variables across the complete data collection period for the whole study population.

**Table 1:** Descriptive statistics of the study population (N = 55)

Time-fixed variables			Mean (SD)	*N* (%)
Demographic data	Age (in years)		34.2 (9.7)	
	Gender	Male		29 (53)
		Female		26 (47)
	Country	Slovenia		26 (47)
		Belgium		29 (53)
Job category		Admin and technical staff		15 (27)
		Researcher without a PhD		26 (47)
		Researcher with a PhD		14 (26)

Time-varying variables			Mean (SD)	*N* (%)
Work location	Non-home ^a^			1493 (66)
	At home			768 (34)
Job demands	[Likert scale: 1–4]		2.2 (0.5)	
Job control	[Likert scale: 1–4]		2.8 (0.4)	
Job strain	Demand/control ratio		0.8 (0.2)	
Social support ^b^	[Likert scale: 1–4]		3.2 (0.6)	
Work interfering with personal life (WIPL)	[Likert scale: 1–5]		2.6 (1.1)	
Personal life interfering with work (PLIW)	[Likert scale: 1–5]		2.2 (1.0)	

Number of observations = 2261

*SD* standard deviation

^a^Non-home = Participants did not work exclusively at home on the questioned day. They either worked partially at home, worked at their office, or worked at a third location

^b^Social support includes supervisor support and co-worker support

Participants indicated to have worked approximately twice as often in non-home locations like either their office, a third location, or they transferred between several locations. About a third of the time participants worked at home (34%). Since higher scores indicate higher interference, we observed that participants experienced higher WIPL (2.6, SD = 1.1) compared to PLIW (2.2, SD = 1.0).

### Inferential results

**Fig. 2:**
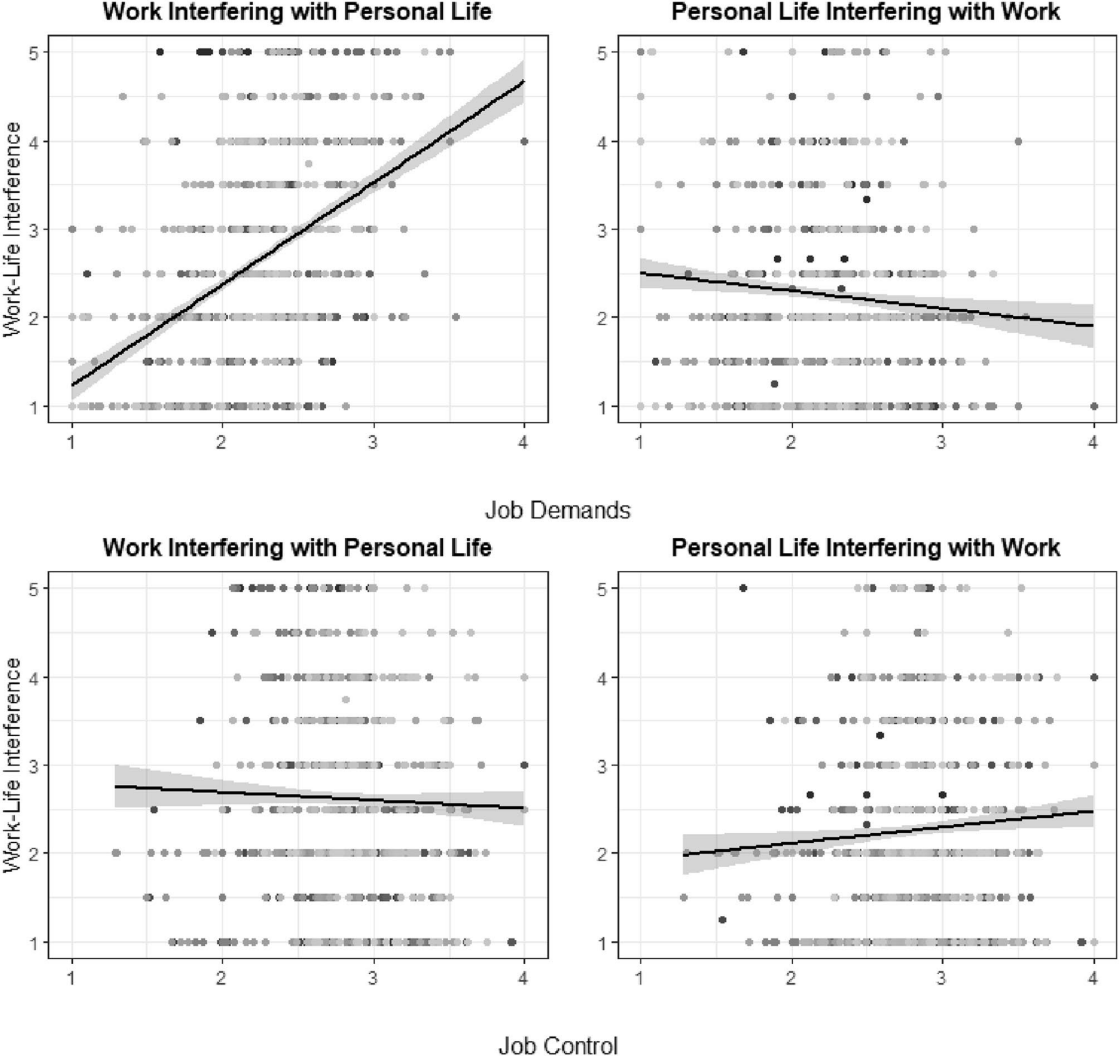
Crude associations of job demands and job control with work interfering with personal life (WIPL) and personal life interfering with work (PLIW). For job demands and job control, items were answered on a 4-point Likert scale ranging from: “*I strongly disagree (1)*” to “*I strongly agree (4)*”. For WIPL and PLIW, items were answered on a 5-point Likert scale ranging from: “*I strongly disagree (1)*” to “*I strongly agree (5)*”

**Fig. 3:**
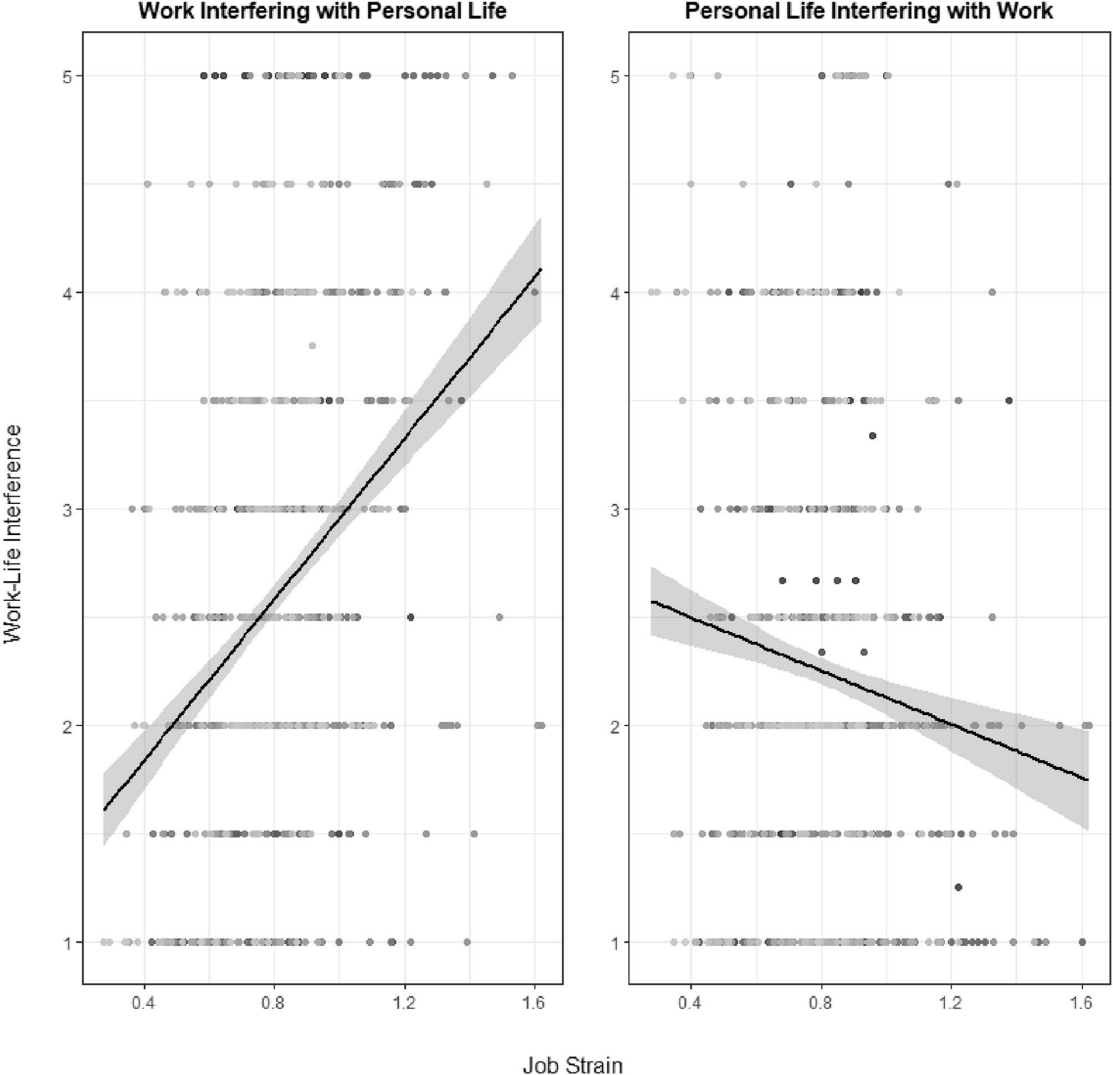
Crude associations of job strain with work interfering with personal life (WIPL) and personal life interfering with work (PLIW). The demand/control ratio was calculated by dividing the daily means of job demands by the daily means of job control. For WIPL and PLIW, items were answered on a 5-point Likert scale ranging from: “*I strongly disagree (1)*” to “*I strongly agree (5)*”

The crude associations of job demands, job control, and job strain with work–life interference are shown in Figs. 2 and 3. The results of the generalized linear mixed models are presented for WIPL in Table 2 and for PLIW in Table 3.

**Table 2:** Random-intercept models of the associations between day-to-day job demands, job control, job strain, and social support and work interfering with personal life (WIPL)

	Fixed-effect regression coefficient (95% CI)						
	Model I	Model II		Model III		Model IV	
		IIa	IIb	IIIa	IIIb	IVa	IVb
Time-fixed variables							
Age	0.02 (− 0.01;0.06)	0.02 (− 0.01;0.05)	0.02 (− 0.01;0.05)	0.02 (− 0.01;0.05)	0.02 (− 0.01;0.05)	0.02 (− 0.01;0.05)	0.02 (− 0.01;0.05)
Gender: female	0.18 (− 0.05;0.42)	0.16 (− 0.06;0.38)	0.19 (− 0.04;0.41)	0.13 (− 0.09;0.35)	0.17 (− 0.06;0.40)	0.12 (− 0.10;0.34)	0.17 (− 0.07;0.40)
Country: Belgium ^a^	** − 0.26 (− 0.51**;− 0.02)*	− 0.23 (− 0.46;0.00)*	− 0.22 (− 0.46;0.01)	− 0.19 (− 0.42;0.04)	− 0.20 (− 0.43;0.04)	− 0.19 (− 0.42;0.04)	− 0.20 (− 0.44;0.04)
Job category: Researcher without a PhD ^b^	− 0.09 (− 0.52;0.33)	− 0.08 (− 0.48;0.33)	− 0.06 (− 0.48;0.35)	− 0.08 (− 0.48;0.32)	− 0.08 (− 0.50;0.33)	− 0.09 (− 0.49;0.31)	− 0.08 (− 0.50;0.33)
Researcher with a PhD	**0.39 (0.04**;0.74)*	0.28 (− 0.06;0.61)	0.35 (0.01;0.69)*	0.28 (− 0.05;0.61)	0.37 (0.02;0.71)*	0.28 (− 0.06;0.61)	0.37 (0.02;0.72)*
Time-varying variables							
Work location: at home ^c^	− 0.01 (− 0.06;0.03)	0.00 (− 0.04;0.05)	− 0.01 (− 0.05;0.04)	0.01 (− 0.05;0.06)	0.00 (− 0.06;0.05)	0.00 (− 0.05;0.05)	0.00 (− 0.06;0.05)
Job demands		**0.47 (0.34**;0.59)***		**0.53 (0.39**;0.67)***		0.48 (− 0.10;1.10)	
Job control		**0.15 (0.02**;0.28)*		**0.19 (0.05**;0.34)**		**1.00 (0.30**;1.60)**	
Job strain (demand/control ratio)			**0.58 (0.30**;0.86)***		**0.61 (0.29**;0.92)***		− 0.19 (− 1.60;1.20)
Social support				− 0.10 (− 0.21;0.01)	− 0.07 (− 0.19;0.04)	0.54 (− 0.11;1.20)	− 0.26 (− 0.60;0.08)
Job demands by social support						0.01 (− 0.16;0.19)	
Job control by social support						** − 0.24 (− 0.44**;− 0.04)*	
Job strain by social support							0.25 (− 0.17;0.66)

Higher scores indicate higher demands, control, strain, support, and interference

*N* = 55; number of observations = 2261, *CI* confidence interval

^*^p < 0.05

^**^p < 0.01

^***^p < 0.001

^a^Ref. Slovenia

^b^Ref. Admin and technical staff

^c^Ref. Non-home: Participants did not work exclusively at home on the questioned day. They either worked partially at home, worked at their office, or worked at a third location

**Table 3:** Random-intercept models of the associations between day-to-day job demands, job control, job strain, and social support and personal life interfering with work (PLIW)

	Fixed-effect regression coefficient (95% CI)						
	Model I	Model II		Model III		Model IV	
		IIa	IIb	IIIa	IIIb	IVa	IVb
Time-fixed variables							
Age	0.00 (− 0.03;0.03)	0.00 (− 0.03;0.03)	0.00 (− 0.03;0.03)	0.00 (− 0.02;0.03)	0.00 (− 0.02;0.03)	0.00 (− 0.02;0.03)	0.00 (− 0.02;0.03)
Gender: female	− 0.01 (− 0.20;0.19)	0.00 (− 0.20;0.20)	− 0.01 (− 0.20;0.19)	0.03 (− 0.17;0.23)	0.02 (− 0.18;0.21)	0.03 (− 0.17;0.23)	0.02 (− 0.18;0.22)
Country: Belgium ^a^	0.06 (− 0.14;0.26)	0.07 (− 0.14;0.27)	0.06 (− 0.14;0.26)	0.09 (− 0.12;0.29)	0.07 (− 0.13;0.27)	0.09 (− 0.11;0.30)	0.08 (− 0.13;0.28)
Job category: Researcher without a PhD ^b^	0.04 (− 0.31;0.39)	0.04 (− 0.31;0.40)	0.04 (− 0.31;0.39)	0.05 (− 0.30;0.40)	0.05 (− 0.29;0.40)	0.05 (− 0.30;0.41)	0.06 (− 0.29;0.40)
Researcher with a PhD	0.04 (− 0.27;0.34)	0.06 (− 0.25;0.37)	0.04 (− 0.27;0.34)	0.06 (− 0.25;0.38)	0.04 (− 0.27;0.34)	0.06 (− 0.25;0.37)	0.04 (− 0.26;0.35)
Time-varying variables							
Work location: at home ^c^	0.05 (0.00;0.10)	0.05 (0.00;0.09)	0.05 (0.00;0.10)	0.04 (− 0.01;0.09)	0.05 (0.00;0.10)	0.04 (− 0.01;0.09)	0.05 (0.00;0.10)
Job demands		− 0.08 (− 0.21;0.05)		− 0.02 (− 0.15;0.12)		− 0.11 (− 0.63;0.41)	
Job control		− 0.16 (− 0.31;− 0.01)*		** − 0.20 (− 0.35**;− 0.04)*		− 0.16 (− 0.85;0.52)	
Job strain (demand/control ratio)			0.01 (− 0.27;0.30)		0.13 (− 0.16;0.43)		0.18 (− 1.00;1.30)
Social support				0.03 (− 0.08;0.15)	0.03 (− 0.09;0.14)	− 0.01 (− 0.75;0.72)	0.04 (− 0.31;0.39)
Job demands by social support						0.03 (− 0.14;0.20)	
Job control by social support						− 0.01 (− 0.23;0.21)	
Job strain by social support							− 0.01 (− 0.40;0.37)

Higher scores indicate higher demands, control, strain, support, and interference

*N* = 55; number of observations = 2261, *CI* confidence interval

^*^p < 0.05

^**^p < 0.01

^***^p < 0.001

^a^Ref. Slovenia

^b^Ref. Admin and technical staff

^c^Ref. Non-home: Participants did not work exclusively at home on the questioned day. They either worked partially at home, worked at their office, or worked at a third location

As presented in models I and II of Table 2, the country of participation showed to have a significant effect, since participants in Belgium reported less WIPL compared to participants in Slovenia (model IIa; β =  − 0.23, *p* < 0.05). This association was not present in the other models. Model I and all further models including job strain as an independent variable showed that researchers with a PhD reported higher WIPL compared to administrative and technical staff (model IVb; β = 0.37, *p* < 0.05). As shown in models II and III, we found significant positive associations between all work-related stressors and WIPL (model IIIa; job demands: β = 0.53, *p* < 0.001, job control: β = 0.19, *p* < 0.01, model IIIb; job strain: β = 0.61, *p* < 0.001). Model IVa showed that there is a significant interaction effect between job control and social support on WIPL. Hence, with each unit increase in social support, the effect of job control on WIPL decreased (β = − 0.24, *p* < 0.05).

As presented in models II and III of Table 3, a significant negative association was found between job control and PLIW (model IIIa; β = − 0.20, *p* < 0.05). However, no significant associations were found between job demands, job strain, or social support and PLIW.

We used the Intraclass Correlation Coefficient (ICC) to calculate the proportion of variance explained by the grouping structure of our study population. The index goes from 0 (clustering provides no information) to 1 (observations in the cluster are identical). The coefficient was higher for WIPL (ICC = 0.55) compared to PLIW (ICC = 0.33). Accordingly, 45% of the total variance in WIPL and 67% of the total variance in PLIW are due to within-person variability.

### Sensitivity analysis

We tested the sensitivity of our model IV results. First, we tested the time effect on day level over 15 days of data collection. Second, we tested the weekend effect to check for differences between Monday and Tuesday versus Wednesday to Friday. However, no significant effects were found, neither for WIPL nor for PLIW.

## Discussion

This is the first study to examine the associations between day-to-day job demands, job control, and job strain and work–life interference among office workers in academia. Additionally, we investigated the differing effect of these work-related stressors on WIPL and PLIW, depending on the levels of social support at work.

Our descriptive results showed that our participants worked about a third of their time at home instead of at the office or a third location. We included the question about work location as a response to the COVID-19 pandemic, which was not part of the original study protocol. The consequences of work-from-home on workers’ productivity and well-being have gained increasing attention in research since the start of the COVID-19 pandemic (Tejero et al. 2021). Our results showed no significant influence of the work location on work–life interference. However, a recent study among office workers has shown that during work-from-home, the social support of colleagues declined, leading to increased stress, resulting in lower productivity and a poorer work–life balance (Tejero et al. 2021). Our study population covered a wide range of occupations and seniority levels among office workers in academia. The results showed that researchers with a PhD reported experiencing significantly higher levels of WIPL compared to administrative and technical staff. Research among US professors during the COVID-19 pandemic showed that assistant and associate professors reported higher work and home stress compared to full professors, which reported moderate work stress and low home stress. Associate professors additionally reported increased workload, stress, and a poorer work–life balance (Kotini-Shah et al. 2022). While these results conflict with our findings, showing higher ranked staff experiencing lower WIPL, another study found similar results to ours, where no significant differences between the seniority levels could be found (O’Laughlin and Bischoff 2005). There might be a curve-shaped trend in which work–life interference increases from early career starters until junior professorship, which then decreases with professorship seniority.

Previous literature found a significant association between work stress and higher work–life interference among female academics compared to male academics (O’Laughlin and Bischoff 2005). However, we did not find a significant difference between the genders.

Our models showed significant associations between all work-related stressors and increased WIPL. Higher job demands and higher job strain are correlated with higher WIPL, which is in line with the Job Demand-Control-Support model (Karasek et al. 1998) and the Work-Family Role Pressure Incompatibility model of Greenhaus and Beutell (1985). The latter suggested that high time devotion to work tasks or pressure experienced at work can lead to work–life interference. A study among US academics confirmed that working overtime due to high job strain can cause work–life interference (O’Laughlin and Bischoff 2005). However, contrary to the theory of the Job Demand-Control-Support model (Karasek et al. 1998) in which low job control is associated with increased stress, our results showed a negative association, meaning higher control was correlated with higher WIPL. This novel insight is consistent with the findings of our focus group study, conducted as a preparatory step for this paper. The participating office workers reported that decision latitude—a subscale of job control of the Job Demand-Control-Support model (Karasek et al. 1998)—was of high relevance to them in their work experiences, providing freedom and the liberty to make their own decisions. However, it was also reported to be very stressful when work limits were not set (Bolliger et al. 2022). A second focus group study confirmed that high responsibility can result in self-doubt, insecurity, or even burnout (Ironside et al. 2019). Perhaps, highly dedicated employees control their job in a way that it interferes with their personal life, for example, by working overtime by their own will. Social support at work proved to be a significant interaction term. It might be interpreted as job control being positively correlated with WIPL, but then being counter-balanced by social support. This result is in line with the Job Demand-Control-Support model (Karasek et al. 1998), presenting social support as a key component and protective factor in work stress experiences.

Job control was also a significant predictor of PLIW. This association is as expected, with higher control being correlated with lower PLIW, confirming the Job Demand-Control-Support model (Karasek et al. 1998). Previous studies suggested that workers who have control and autonomy over how, where, and when they work can arrange for an effective and positive work–life balance (Kalliath and Brough 2008). Results of another study among office workers are in line with ours, suggesting that high levels of flexibility and control were associated with a healthy work–life balance (Bjärntoft et al. 2020). A similar concept is the self-perceived boundary control. This concept confirmed our results by suggesting that having control over work and private life boundaries contributes positively to a healthy balance between work and one’s personal life (Kossek et al. 2012).

### Strengths and limitations

The main strength of this study is the detailed and sophisticated data collection procedure by having developed an EMA embedded in our STRAW smartphone application (Lukan et al. 2020). The collected data provided us with a large dataset of repeated measurements including 55 workers in academia across 15 working days. These repeated measurements enabled us to research day-to-day work stress experiences, compared to traditional studies looking into chronic stress. Furthermore, despite a highly demanding data collection protocol, we had a very strong participant adherence with no drop-outs between briefing and debriefing and only 5.2% missing data points. Another strong aspect is the increasing relevance of work–life balance in occupational research and practice. The rising attention can be justified by increasing concerns for workers’ quality of work life, a growing number of two-income households, and increasing expectations of self-fulfillment through work (Greenhaus and Beutell 1985). This seems particularly relevant, since the COVID-19 pandemic had a major influence on the changing work environments of office workers.

Our recruitment strategy applying a convenience sampling method comes with limitations. Using a non-random sample can introduce selection bias. We need to mention here that we most likely included workers with, first, an intrinsic interest in the topic of work stress and, second, the capacity to participate in our study. A possible consequence of this is limited external validity toward other (non-academic) office jobs. A further point to discuss is the selection of questionnaire items per EMA. Based on the triggering protocol, two items per questionnaire (subscale) were randomly chosen for each EMA to keep them concise and to stimulate a variation of the items. This innovative approach allowed us to include many dimensions of work stress per EMA without too much burden for the participants. However, no studies were available to refer to concerning the external validity and reliability of such a method. Further research is needed to advice on the development of similar studies.

### Conclusion

Based on our EMA study, higher day-to-day job demands and job strain were significantly correlated with higher day-to-day WIPL. Interestingly, day-to-day job control was also a significant predictor of higher day-to-day WIPL, contrary to what other studies and well-established work stress models suggest. On the other hand, day-to-day job control was significantly correlated with lower day-to-day PLIW, which is in line with previous research. Additionally, we found a significant interaction effect between job control and social support at work on WIPL. Based on our results, WIPL proved to be a more relevant work stress outcome for office workers in academia, compared to PLIW.

These results urged us to draw two main conclusions. First, for further research, we recommend also considering WIPL and PLIW as two different concepts. Second, for stress prevention approaches, office workers in academia should be supported to balance their job control to be able to keep the work–life interference to a minimum and practice a healthy work–life balance.

## Data Availability

The datasets generated during and/or analyzed during the current study are available from the corresponding author upon reasonable request.
